# Identification and Expression of the CorA/MRS2/ALR Type Magnesium Transporters in Tomato

**DOI:** 10.3390/plants12132512

**Published:** 2023-06-30

**Authors:** Wen Liu, Shahbaz Khan, Mengying Tong, Haiyan Hu, Liyan Yin, Jiaquan Huang

**Affiliations:** 1 Hainan Key Laboratory for Sustainable Utilization of Tropical Bioresource, College of Tropical Crops, Hainan University, Haikou 570228, China184114@hainanu.edu.cn (S.K.); tongmy_07@163.com (M.T.); yanhai0987@163.com (H.H.); 2 Hainan Key Laboratory for Sustainable Utilization of Tropical Bioresources, School of Life Sciences, Hainan University, Haikou 570228, China; lyyin@163.com

**Keywords:** magnesium transporter, qRT-PCR, RT-PCR, tomato (*Solanum lycopersicum* L.), genomic analysis

## Abstract

Magnesium (Mg^2+^) is the most abundant divalent ion in plants, participating in numerous metabolic processes in growth and development. CorA/MRS2/ALR type Mg^2+^ transporters are essential for maintaining Mg^2+^ homeostasis in plants. However, the candidate protein and its potential functions in the tomato plant have not been fully understood. In this study, we identified seven MGT genes (*SlMRS2*) in tomato based on sequence similarity, domain analysis, conserved motif identification, and structure prediction. Two *SlMRS2* genes were analyzed in the bacterial strain MM281, and a functional complementary assay demonstrated their high-affinity transport of Mg^2+^. Quantitative real-time PCR analysis revealed that the expressions of these Mg^2+^ transporters were down-regulated in leaves under Mg^2+^ limitation, with a greater impact on lower and middle leaves compared to young leaves. Conversely, under Mg^2+^ toxicity, several genes were up-regulated in leaves with a circadian rhythm. Our findings indicate that members of the SlMRS2 family function as Mg^2+^ transporters and lay the groundwork for further analysis of their distinct functions in tomato.

## 1. Introduction

Magnesium (Mg^2+^) is the most abundant divalent cation in plant cells and the second most abundant cation after potassium. As the center atom of chlorophyll, it is essential for chlorophyll synthesis and degradation [1]. Mg^2+^ plays an important role in energy metabolism because ATP only has biological activity in conjugated form with Mg, and Mg-ATP accounts for about half of the total Mg^2+^ content in plant cells [2,3]. In addition, Mg^2+^ participates in the activities of more than 300 enzymes involved mainly in signal transduction, secondary metabolism, the synthesis of nucleic acids and proteins, and the bridging of ribosomes during translation [1,2,4]. Therefore, the content of Mg^2+^ in plant cells and organelles is strictly controlled to ensure normal physiological and metabolic processes [4], for which Mg^2+^ transporters have a vital role in controlling the influx and efflux of Mg^2+^ and maintaining Mg homeostasis [1].

In the bacteria, three kinds of Mg^2+^ transporters operated together, including CorA, MgtA, and MgtB; among them, CorA had the highest affinity for Mg^2+^ and is located on the membrane in prokaryotes [5]. The CorA-like protein ALR1 of yeast controlled the transport of approximately 60% of the total amount of Mg^2+^ [6,7]. Another *CorA* homologous *MRS2* gene has been identified in yeast, and its product Mrs2p is an integral protein of the inner mitochondrial membrane [8]. Because of the sequence variability with CorA proteins, they were placed into a separate protein subfamily, MIT (metal ion transporter), which belonged to the CorA superfamily [9,10]. All these proteins transporting Mg^2+^ are collectively called Mg^2+^ transporters (MGT). According to the study by Knoop et al. (2005) [11], the major features of the CorA family are two transmembrane domains (TMs) with the GxN motif (2-TM-GxN) near the C-terminal. At the same time, all CorA/MRS2/ALR type Mg^2+^ transporters share a conserved tripeptide Gly-Met-Asn (GMN) motif on the penultimate TM.

In higher plants, CorA/MRS2/ALR-type Mg^2+^ transporters play vital roles in both Mg^2+^ transport and homeostasis [1,4]. Under Mg^2+^ deficiency conditions, Mg^2+^ accumulates in the roots via mass flow and is absorbed via cell-membrane-located Mg^2+^ transporters, such as AtMGT1/AtMRS2-10, AtMGT6/AtMRS2-4, and AtMGT7/AtMRS2-7 in *Arabidopsis* [12,13,14], and their homology proteins OsMGT1 in rice [15] and ZmMGT12 in maize [16]. AtMRS2-4 and AtMRS2-7 contribute greatly to the adaption of both low and high Mg^2+^ concentrations [17], and AtMRS2-4 also transported Mg^2+^ upward into the blade when there was excess Mg^2+^ in the external environment [18].

Excessive Mg^2+^ could be stored in the vacuole via the transport system located on the tonoplast to maintain the cytoplasmic homeostasis of Mg^2+^, and three Mg^2+^ transporters, AtMGT2/AtMRS2-1, AtMGT3/AtMRS2-5, and AtMHX, participated in this process [19,20]. AtMGT10/AtMRS2-11 is localized on the chloroplast envelope and controlled the bidirectional transfer of Mg^2+^ between chloroplast stroma and the cytoplasm, mainly in vascular tissues [12,21,22], while AtMGT5/AtMRS2-6 is localized on the tapetum membrane and controls the bidirectional transportation of Mg^2+^ between the ovary and the tapetum [23]. Mg^2+^ transporters also played a role in the development of organelles and reproductive organs. *MGT10/AtMRS2-11* is essential for the tissue-specific regulation of chloroplast development [24], and its mutation (*mgt10*) caused the loose stacking of thylakoid membranes in the chloroplast, lessening the light protection and reducing the repair of the damaged PSII [22]. The expression of *AtMRS2-11* was induced by light with circadian rhythm [22,25]; a similar characterization was also found for *ZmMGT12* [26], *SsMGT6*, *SsMGT9*, and *SsMGT10* [27]. In addition, the *AtMGT4/AtMRS2-3*, *AtMGT5/AtMRS2-6*, and *AtMGT9/AtMRS2-2* played important roles in pollen development. Among these, AtMRS2-3 is located on the endoplasmic reticulum and mainly acts during the pollen development stage from double cells to maturity, while the pollen in the *Atmgt4* mutant was inactivated in the double cell phase [28]. *AtMRS2-6* was essential for the male gamete mitosis and inner wall formation [23]. The function of *AtMRS2-2* has been found essential for pollen development, while pollen grains were aborted in deletion mutants [29]. *PbrMGT7* was also expressed in the pollen, and acted on the mitochondria to keep the homeostasis of Mg^2+^ in pollen development [30]. Mg^2+^ transporters also alleviate the aluminum (Al) toxicity of plants. AtMRS2-1, located on the vacuole, showed no sensitivity to the Al stress, while AtMRS2-10 and AtMRS2-11 exhibited high sensitivity to the Al toxicity [31]. Similarly, OsMGT1 improved Al tolerance by enhancing the concentration of Mg^2+^ in rice cells [32].

Although much work has been conducted on Mg^2+^ transporters, to our knowledge no systematic work has been carried out on tomato (*Solanum lycopersicum* L., 2n = 24). Tomato is an important cash crop and vegetable in China, and the yield and consumption of tomato are increasing rapidly [33]. Moderate to severe magnesium deficiency frequently occurs in the field [34], and it could lead to a 40–60% yield reduction. The elucidation of the function of Mg^2+^ transporter genes in tomato would be of great help to reduce Mg^2+^ deficiency via exploring the internal genetic potential. In this study, seven CorA/MRS2/ALR-type Mg^2+^ transporters were identified to constitute the typical Mg^2+^ transport system of tomatoes. The biochemical information, phylogenetic relationships, sequence characteristics, gene distribution, and collinearity were analyzed, and protein quaternary structures were predicted via bioinformatics technology. A complementation assay was used to verify the Mg^2+^ transport function of *SlMRS2*. Also, the expression profiles of *SlMRS2* genes, including their expression patterns and responses to Mg^2+^ stress, are shown in this article.

## 2. Results

### 2.1. Identifications of Mg^2+^ Transporters

**Figure 1:**
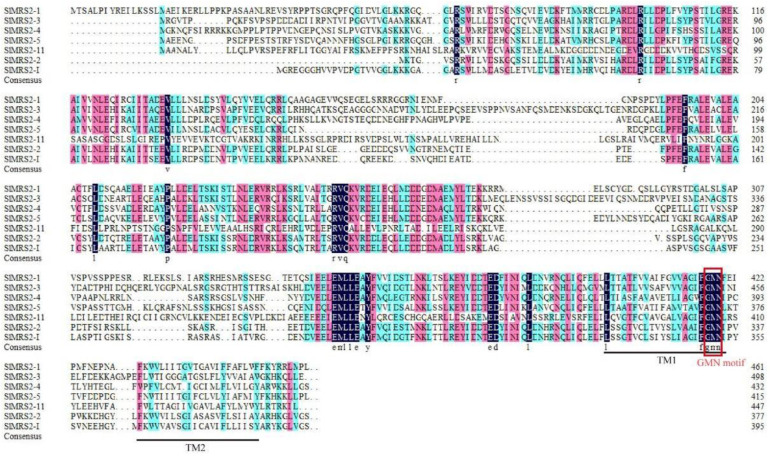
Multiple sequence alignment analysis of Mg^2+^ transporters in tomato. The alignment map was prepared using DNAMAN, wherein the same sequence was marked black, sequence similarity of more than 75% was marked as rose, sequences less than 50% were colorless, and the remaining sequence was marked cyan. Two conserved transmembrane domains were marked with black lines, and a red box was used to indicate the conserved tripeptide GMN.

Seven Mg^2+^ transporters of tomato were detected via blast using the Mg^2+^ transporter sequence of the model plants as a query. Bioinformatics analysis indicated that the isoelectric points of Mg^2+^ transporters in tomato ranged from 4.56 to 6.09, and the relative molecular mass was between 42.53 and 54.98 kDa (Table 1). The predicted transmembrane domains and the conserved tripeptide GMN (Gly-Met-Asn) suggested that these proteins were Mg^2+^ transporters (Figure 1). Subcellular location prediction showed that SlMRS2-1, SlMRS2-5, and SlMRS2-11 were located on the plasma membrane, and SlMRS2-2 and SlMRS2-3 were located on the chloroplast membrane, SlMRS2-I on the tonoplast, and SlMRS2-4 located both on the nucleus and plasma membrane. The diverse subcellular localization of these multiple members indicated that they might coordinate to maintain intracellular Mg^2+^ homeostasis in tomato.

### 2.2. The phylogenetic Tree Analysis

**Figure 2:**
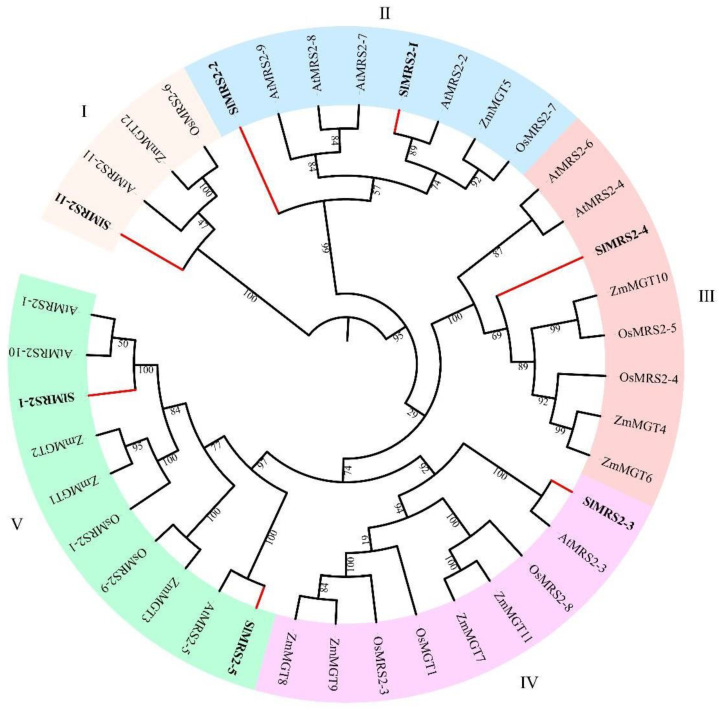
The phylogenetic tree of Mg^2+^ transporters of tomato, maize, rice, and *Arabidopsis*. The neighbor-joining tree was contrasted using Mega X. These Mg^2+^ transporters were divided into 5 clusters (I–V), in different colors. The red branches represent Mg^2+^ transporters in tomato with the prefix Sl.

The peptide sequences of Mg^2+^ transporters, obtained from tomato variety Heinz 1706 (the whole genome was sequenced), *Arabidopsis thaliana*, maize, and rice, were used to build the phylogenetic tree (Figure 2). A total of 39 proteins from four species were divided into five clusters. SlMRS2-3 was presented in cluster IV and the SlMRS2-1, 2-5 belonged to cluster v. SlMRS2-2 and 2-1 were in cluster II, and SlMRS2-4 was a member of cluster III. Only SlMRS2-11 belonged to cluster I. Most of the Mg^2+^ transporters in tomato were more similar to the counterpart sequences from *Arabidopsis* compared to those from maize and rice. Based on the sequence similarity and the constructed phylogenetic tree, it was probable that *SlMRS2-11* was an *AtMRS2-11* homologous gene, *SlMRS2-3* was an *AtMRS2-3* homologous gene, *SlMRS2-5* was an *AtMRS2-5* homologous gene and *SlMRS2-1* was an *AtMRS2-1* homologous gene. No homologous genes were found for the other three genes from tomato.

### 2.3. The Gene Structure, Conserved Motifs, and Cis-Acting Elements

**Figure 3:**
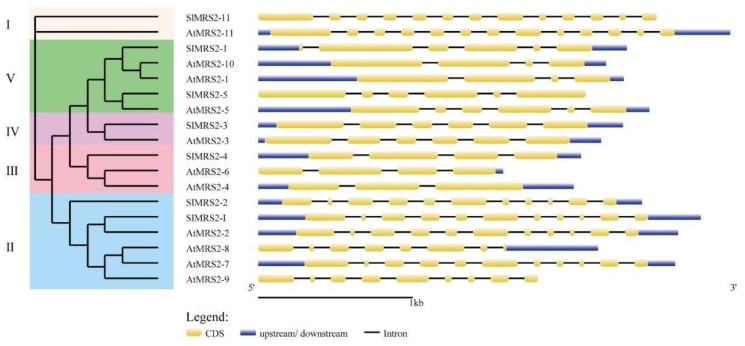
Gene structure analysis of Mg^2+^ transporters in tomato and *Arabidopsis* according to their phylogenetic relationship. All sequences were classified into 5 groups with different colors (**left**). The yellow boxes represent exons, the thin solid black lines represent introns, and the blue boxes indicate upstream or downstream sequences (**right**).

**Figure 4:**
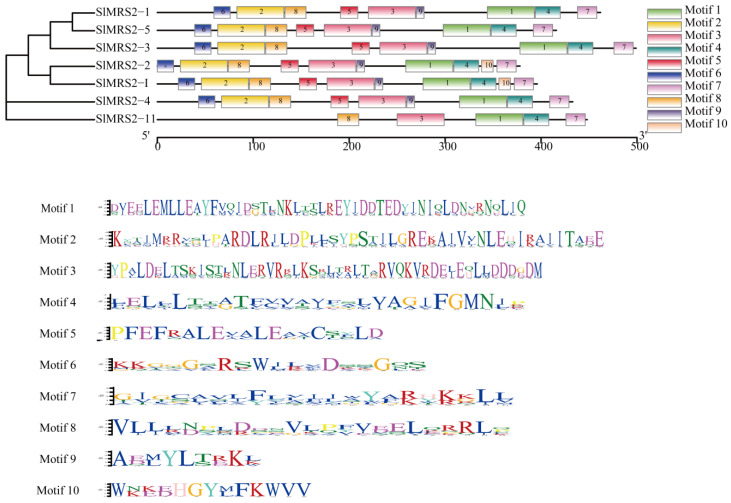
Conserved motifs in tomato Mg^2+^ transporters. The phylogenetic tree and the motif distribution are shown. A total of 10 motifs were detected. The detailed peptide sequences of each motif are also shown.

The gene structures in both tomato and *Arabidopsis* exhibited large differences in the numbers of exons between different clusters (Figure 3). The number of exons in the sequences varied from 3 to 11, and the largest number of exons was observed in cluster I, while the smallest number was detected in cluster III. This result also confirmed the evolutionary relationship between the candidate members of Mg^2+^ transporters in tomato. Conserved motifs of potential Mg^2+^ transporters are displayed in (Figure 4). Motifs 1, 3, 4, 7, and 8 existed in all tested sequences, and only the short peptide EMLLE in motif 1, RVQ in motif 3, and FGMN in motif 4 were identical. Motif 10 was unique to genes *SlMRS2-2* and *SlMRS2-I* in cluster II. *SlMRS2-11* lacked motifs 2, 5, 6, and 9. Among these motifs, 1, 4, and 7 in the CorA domain might contribute greatly to the function of these genes, and conserved tripeptide GMN in motif 4 near the C-terminal was one of the symbols of CorA/MRS2/ALR type Mg^2+^ transporter.

**Figure 5:**
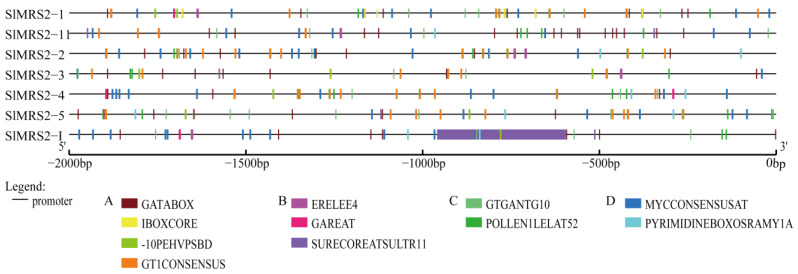
Cis-acting element prediction of tomato Mg^2+^ transporter genes. These cis-acting elements were analyzed using PLACE based on the sequence 2000 bp upstream of the initiation codon. A total of 11 cis-acting elements are shown here, and these elements were divided into 4 types. A: light-induced elements; B: plant hormone regulatory elements; C: pollen developmental elements; D: dehydration response elements.

The cis-acting element prediction indicated four types of regulatory units found in seven genes (Figure 5), including light-induced elements and plant hormone regulatory, pollen developmental, and dehydration-responsive elements. Some elements appeared more than once in the same sequence, and the elements belonging to light-induced units were the most abundant. This might indicate that the expression of Mg^2+^ transporters in tomato is controlled by multiple conditions and, most importantly, by light.

### 2.4. Chromosomal Location and Gene Distribution

**Figure 6:**
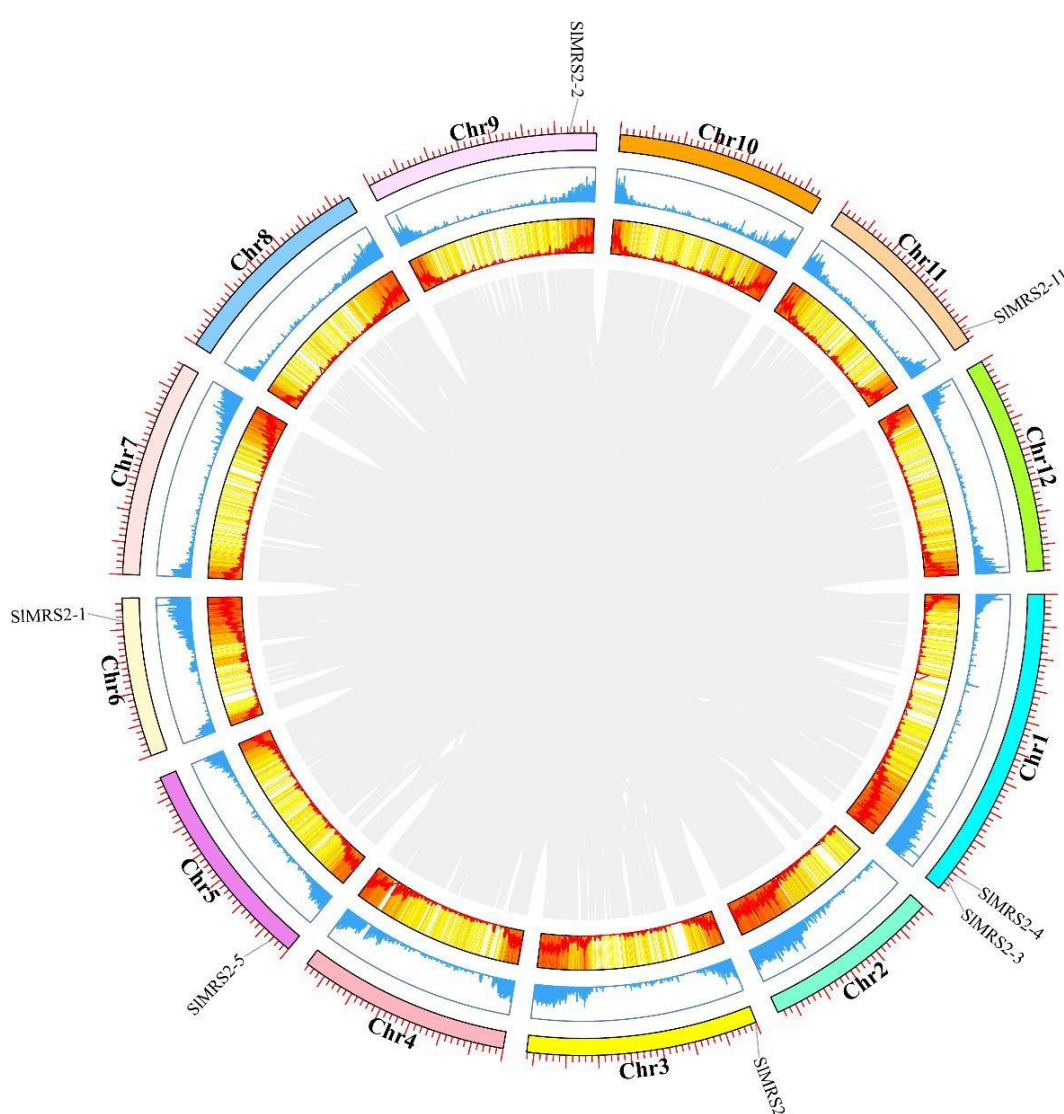
Chromosomal location, gene distribution density, and interchromosomal relationships of *SlMRS2* genes. The innermost showed the collinear analysis of the tomato genome. The middle circles showed the gene distribution density in the form of heat map, and the outermost circle showed the locations of Mg^2+^ transporters on different chromosomes of tomato.

Mg^2+^ transporters in tomato are located on chromosomes 1, 3, 5, 6, 9, and 11, and all of them were in a region of the chromosome where genes were densely distributed (Figure 6). According to the collinearity analysis, tomato Mg^2+^ transporter genes were not under high evolution selection pressure, and their non-redundant characteristics might enable this crop to be a model plant to functionally analyze the Mg^2+^ transporter genes.

### 2.5. Three-Dimensional Structure Prediction of Magnesium Transporter

**Figure 7:**
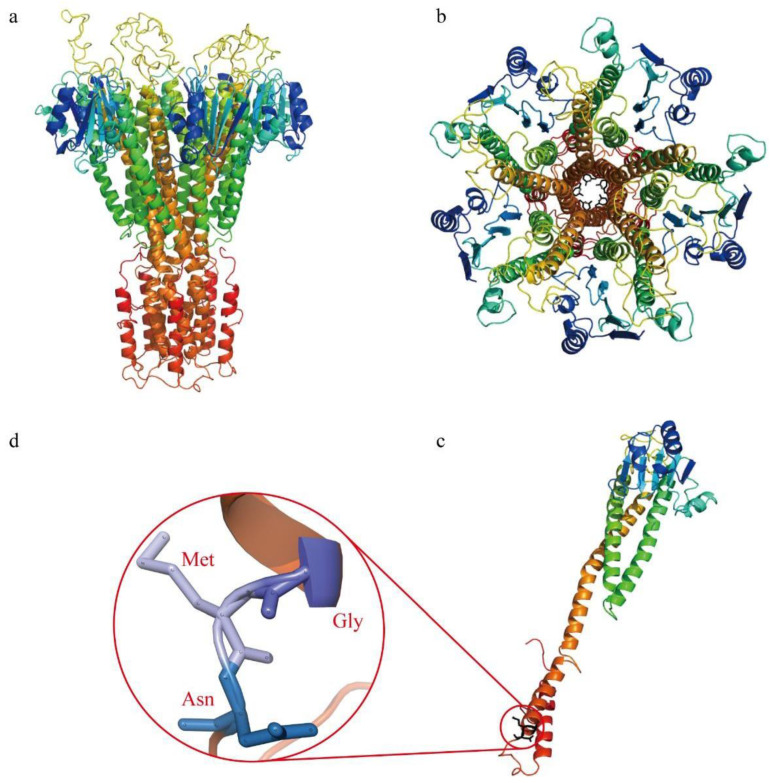
Spatial structure prediction of SlMRS2-11. (**a**) Side view of protein complex; (**b**) vertical view of protein complex; (**c**) the structure of the monomer and location of conserved tripeptide GMN; (**d**) the condensed structural formulas of GMN.

All Mg^2+^ transporter proteins had similar spatial structures, so in this study only SlMRS2-11 were presented (Figure 7). The integrated 3D structure of SlMRS2-11 is composed of five monomers (Figure 7a,b), each monomer is composed of five helixes (Figure 7c), and the conserved tripeptide motif GMN is located on the C-terminal (Figure 7d). As a result, a channel resembles a five-pointed star, with the tripeptide GMN pointing to the center of the channel. The unique structure of the protein and the position of GMN might determine its specificity to transport Mg^2+^ and other metal ions.

### 2.6. Expression Analysis of SlMRS2 Genes in Tomato

**Figure 8:**
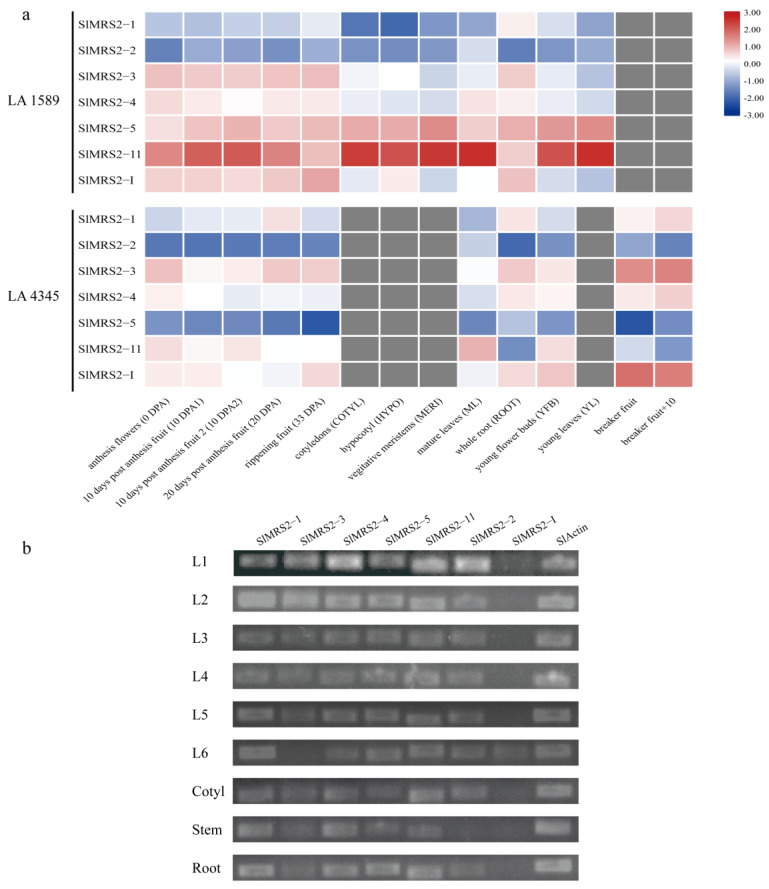
The expression pattern of Mg^2+^ transporters in tomato. (**a**) Heatmap of Mg^2+^ transporter gene expression in different parts of tomato cultivars LA1589 and LA4345; (**b**) gel electrophoresis of semi-quantitative RT-PCR analysis of Mg^2+^ transporter genes in leaves, cotyledon, stem, and root of tomato. L1–L6 indicated leaves from full maturity to youngest.

The heatmap of the expression of seven Mg^2+^ transporter genes was drawn using the RNAseq data in two tomato cultivars (Figure 8a). The results demonstrated a contrasting expression pattern between two varieties for a specific gene. At the same time, great expression variation was also detected in different tissues. For example, *SlMRS2-11* was mainly expressed in mature leaves, while *SlMRS2-1* showed high expression in the roots of both varieties. The semi-quantitative PCR results using tomato variety Qianxi also indicated that *SlMRS2* genes were expressed in almost all tissues, with some exceptions; *SlMRS2-3* was not expressed in the youngest fully expanded leaf, *SlMRS2-2* was not expressed in the stem, and *SlMRS2-I* was only expressed in the fully expanded leaf (Figure 8b). It is suggested that *SlMRS2* genes were expressed cooperatively, while some members were strictly controlled and closely related to the tomato variety.

**Figure 9:**
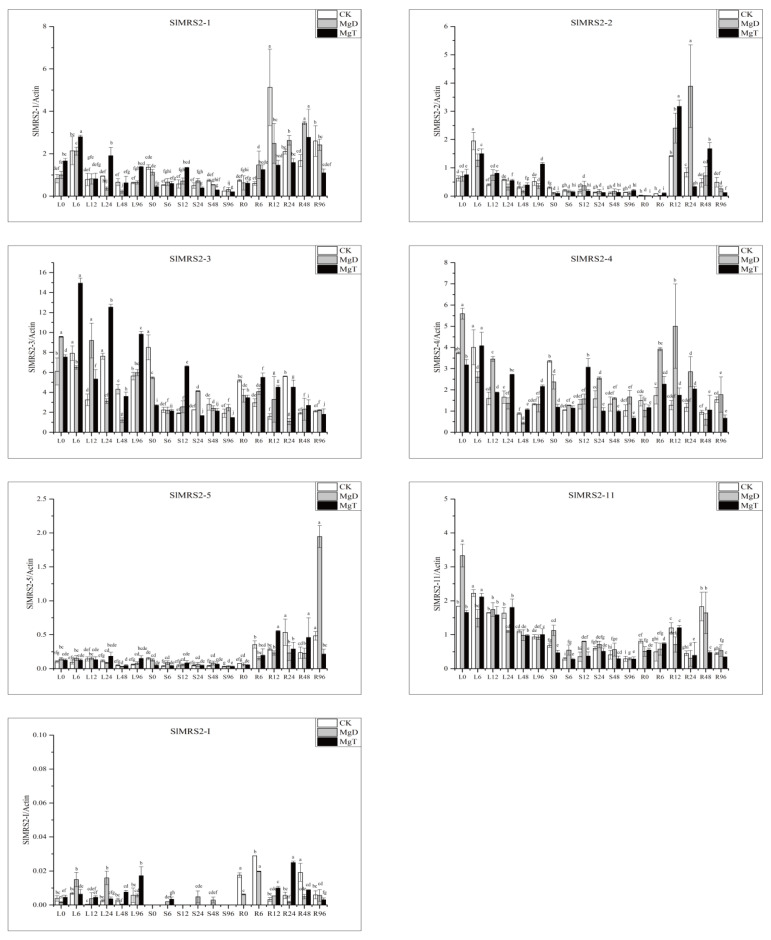
The expression of *SlMRS2* genes in the leaves, stems, and roots of tomato after 0, 6, 12, 24, 4, and 96 h of Mg^2+^ treatment. L: leaves; S: stem; R: root. CK: 1 mM Mg^2+^ as a control; MgD: 0 mM Mg^2+^ as Mg^2+^ deficiency; MgT: 5 mM Mg^2+^ as Mg^2+^ toxicity. The *actin* gene was used as an internal control to normalize the expression data, and the error bars represent ±SD (n = 3). Different letters on bars indicate significant differences using Tukey’s HSD test at *p* ≤ 0.05.

The tissue and time-dependent expression characterization of Mg^2+^ transporter genes under different Mg^2+^ treatments is displayed in Figure 9. The Mg^2+^ toxicity and deficiency did not affect the tissue-specific gene expression. The tissue expression revealed that all seven genes were expressed in all three tested tissues, but most of the Mg^2+^ transporter genes were more expressed in leaves and roots as compared to shoots. *SlMRS2-I* was hardly detected in stems, and its expression level was also low in other tissues. Higher expressions of *SlMRS2-1*, *2-2*, *2-3*, *2-4*, and *2-11* were detected in leaves and roots when compared with stems, while *SlMRS2-5* was mainly expressed in roots.

The time response showed that most genes expressed in the root increased first and then decreased under Mg^2+^ stress conditions (Figure 9), but the time of peak expression differed slightly among different members. For example, the expression of *SlMRS2-1* peaked in roots at 48 h under both low and high Mg^2+^ conditions, while *SlMRS2-3* and *SlMRS2-4* reached their peak levels at 6 h and 12 h, respectively. In leaves, the gene expression tended to decrease under Mg^2+^ deficiency. At the same time, several genes, such as *SlMRS2-1*, *2-3*, *2-4*, and *2-11*, showed circadian rhythms in gene expression under high Mg^2+^.

**Figure 10:**
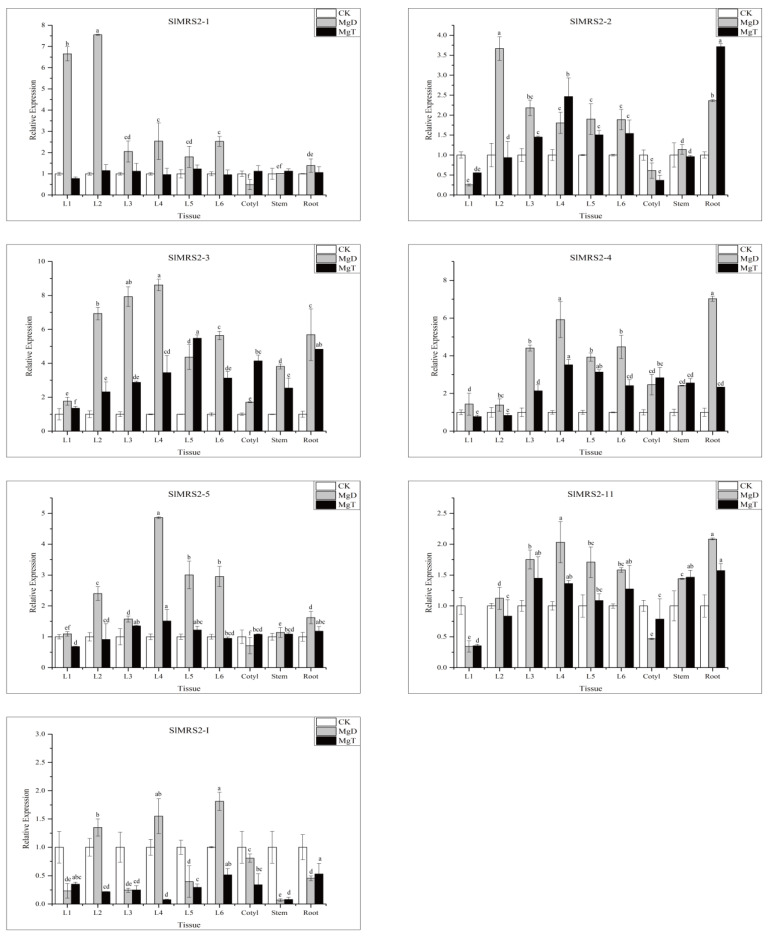
Effects of Mg^2+^ stress on the relative expression of *SlMRS2* genes in leaves, cotyledon, stem, and root of tomato. L1–L6 indicated leaves from fully mature to youngest. CK: 1 mM Mg^2+^ as a control; MgD: 0 mM Mg^2+^ as Mg^2+^ deficiency; MgT: 5 mM Mg^2+^ as Mg^2+^ toxicity. The *actin* gene was used as an internal control to normalize the expression data, and the error bars represent ±SD (n = 3). Different letters on bars indicate significant differences using Tukey’s HSD test at *p* ≤ 0.05.

The relative expressions of Mg^2+^ transporter genes were analyzed in roots, stems, cotyledons, and leaves from different positions of tomato after 6 days of Mg^2+^ treatment (Figure 10). All *SlMRS2* genes in the tested tissues were up-regulated under the Mg^2+^ deficiency condition, except the expressions of *SlMRS2-I*, *SlMR2-2*, *SlMR2-5*, *SlMR2-11*, and *SlMR2-I* in cotyledon, *SlMRS2-2* and *SlMR2-11* in leaf 1, and *SlMR2-I* in leaves 1, 3, and 5. Under excessive Mg^2+^ conditions, the expression of *SlMRS2-1* and *SlMRS2-5* remained unchanged, and the expression of *SlMRS2-2, 2-3*, *2-4*, *2-5*, and *2-11* was correlated with leaf age, especially from the third to the fifth expanded leaf (L2–L4). There were fairly high up-regulated expression levels of *SlMRS2-2*, *SlMRS2-3*, and *SlMRS2-4* in roots.

### 2.7. Function Complementation Analysis of SlMRS2 Genes

**Figure 11:**
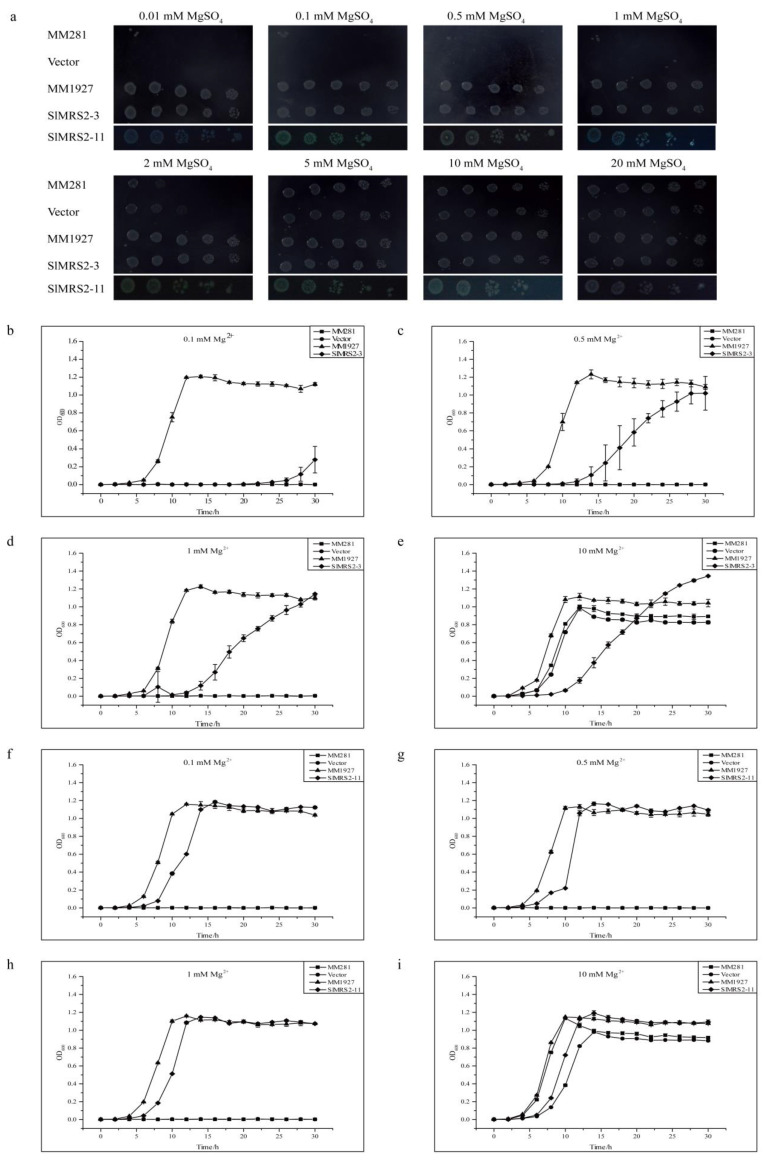
Complementation of *Salmonella typhimurium* mutant MM281 by *SlMRS2-3* and *SlMRS2-11*. The mutant MM281 transformed with an empty vector was taken as negative control and wild strain MM1927 as a positive control. (**a**) Complementation assay on solid medium containing 0.01, 0.1, 0.5, 1, 2, 5, 10, and 20 mM MgSO_4_. The horizontal lines show 10-fold dilutions of bacterial culture in a series. (**b**–**e**) Growth curve of MM281 strain expressing *SlMRS2-3* grown on the liquid medium containing 0.1, 0.5, 1, and 10 mM MgSO_4_. (**f**–**i**) Growth curve of MM281 strain expressing *SlMRS2-11* grown on the liquid medium containing 0.1, 0.5, 1, and 10 mM MgSO_4_. Growth was monitored every 5 h as OD_600_. The error bars represent ±SD (n = 3).

A total of six *SlMRS2* genes (*SlMRS2-1*, *2-3*, *2-4*, *2-5*, *2-11*, *2-I*) from the tomato cultivar Qianxi were cloned and sequenced. They were then reconnected into the pTrc99A vector and transferred into MM281 to grow in media with different Mg^2+^ concentrations. The results indicated that only *SlMRS2-3* and *SlMRS2-11* restored the growth of MM281 on the medium containing low Mg^2+^ concentration, and these two genes had relatively high affinities to Mg^2+^, so that the transformed MM281 could grow on the medium with 0.01 mM Mg^2+^ (Figure 11a). The growth curves in the liquid media reconfirmed the results from the solid media (Figure 11b–i). *SlMRS2-3* had a significant retarding effect on the growth of the strain when compared to that of *SlMRS2-11*, indicating that *SlMRS2-3* might have a lower ability to transport Mg^2+^ than *SlMRS2-11*. Unfortunately, the other tested genes showed no complementary effect under present conditions.

## 3. Discussion

Mg^2+^ transporters play important roles in maintaining the homeostasis of Mg^2+^ to ensure the normal functioning of various physiological metabolic processes [2]. In this study, seven Mg^2+^ transporters of the CorA family were identified in tomato and the length, molecular mass, and pI values of proteins were predicted. All of the sequences had the CorA domain and conserved tripeptide GMN, which were according to the family characteristics (Table 1, Figure 1). At the same time, the sequence alignment and motif analysis revealed another motif, EMLLE, in the front of the GMN tripeptide motif (Figure 1 and Figure 4), and a similar motif has been reported in *Arabidopsis*, maize, and rice with the conserved tripeptide LLE [12,35,36]. Thus, we inferred that EMLLE is another symbolic conserved motif of CorA/MRS2/ALR type Mg^2+^ transporters in plants. The spatial structure prediction of the Mg^2+^ transporter showed that it is a protein complex composed of five subunits with a distinct structure in the center of the protein for the Mg^2+^ to pass through the complex (Figure 7), so we guessed that the CorA/MRS2/ALR type Mg^2+^ transporters are ion channel proteins. The glycine-methionine-asparagine (GMN) motif of the TM1 helices was the key structure used to identify the CorA/MRS2/ALR superfamily (Figure 7c), and binds with hydrated Mg^2+^, as predicted by Payandeh et al. (2013) [37]. Based on the previous conclusions that most Mg^2+^ transporters only controlled the influx of Mg^2+^ [1,4], we suggested that the C-terminal transmembrane of the Mg^2+^ transporters was anchored in the membrane, and the N-terminal was exposed to the interior of organelles or cytoplasm, as suggested by Lunin et al. (2006) [38].

The Mg^2+^ transporters identified in tomato were significantly less than those in other model plants; for instance, there are 11 MGTs in *Arabidopsis*, 9 in rice, and 12 in maize [35,36,39]. The possible reason might be that the tomato genome acts as a low-copy genome, with fewer gene duplications and fewer high-frequency copy sequences, and this genome composition is rare in angiosperms [40,41]. Therefore, it was not surprising to find that some MGTs in *Arabidopsis*, rice, and maize had no homologous proteins in tomato (Figure 2). It might be possible that some other proteins, such as the *NIPA* (Nonimprinted in Prader-Willi/Angelman syndrome) gene family, which is rarely studied in plants, are involved in tomato [42,43]. Such proteins might transport Mg^2+^ to supplement the functional defects of CorA family proteins in some transport processes, or possibly the Mg^2+^ transporters of the CorA family in tomato are non-redundant and possess functions of several transporter genes in other plants. This non-redundant characteristic might be of great merit to analyze the function of specific Mg^2+^ transporters in tomato.

In this study, many cis-acting elements, such as the plant hormone regulatory element, dehydration response elements, light response elements, and pollen developmental elements, were presented in the promoter regions of tomato Mg^2+^ transporter genes (Figure 5). Previous studies also showed that several hormones like ethylene, auxin, gibberellin, and abscisic acid responded to Mg^2+^ stress in *Arabidopsis* and induced downstream metabolism, including photoprotection and antioxygen systems [44]. Ethylene interacted with auxin under Mg^2+^ deficiency [45], while abscisic acid and gibberellin tended to respond to Mg^2+^ toxicity [46,47,48]. In addition, the expression of *AtMRS2-11* followed a circadian rhythm through the existence of light response elements [25]. It can thus be inferred that the expression pattern and function of tomato Mg^2+^ transporter are similar to their counterparts in *Arabidopsis*.

The expression of seven *SlMRS2* genes greatly varied in different varieties and tissues (Figure 8). However, no clear studies have shown that the expression of the Mg^2+^ transporter genes in other species was related to the variety. Meanwhile, expression analysis showed that genes with similar functions and a high degree of sequence homology had similar expression profiles. Based on the expression profile and the phylogenetic relationship, the possible function of different genes could be predicted, which might pave the way for the characterization of the candidate genes. For example, *SlMRS2-1* was highly expressed in roots and leaves, while *SlMRS2-5* was mainly expressed in the roots. A similar expression pattern was also reported in homologous genes in *Arabidopsis* (*AtMRS2-1* and *2-5*) [13]. Based on the study showing that *AtMRS2-5* is expressed in the veins of *Arabidopsis* seedlings, it could be speculated that *SlMRS2-5* might mainly control the content of Mg^2+^ in the vacuoles of root cells, and transport Mg^2+^ from vein cells to the extracellular fluid of mesophyll cells in the leaves.

Although Mg^2+^ deficiency showed obvious leaf age differentiation, only *SlMRS2-5* in young leaves and *SlMRS2-1* in old leaves responded to Mg^2+^ deficiency. In particular, *SlMRS2-3* in tomato might be involved in the long-distance transport of Mg^2+^ in tomato seedlings. Previous studies have shown that the function of AtMRS2-3, which is the homologous protein of SlMRS2-3, was related to the development of pollen at the two-cell stage [28]. But the present study showed that the expression of *SlMRS2-3* was consistent with *SlMRS2-4* and both showed a similar expression in various tissues of tomato seedlings. The high and low Mg^2+^ concentrations induced the expression levels of *SlMRS2-3* and *SlMRS2-4* in all tested tissues and showed a more than twofold increase relative to the normal Mg^2+^ level (Figure 9 and Figure 10), while the expression pattern of *AtMRS2-4*, a homologous protein of *SlMRS2-4*, was similar to our study [14,18]. Therefore, it could be speculated that *SlMRS2-3* and *SlMRS2-4* might be involved in absorbing Mg^2+^ in roots and transporting it upward. However, differences were reported in their expression, as *SlMRS2-3* showed more response to a high Mg^2+^ environment, while *SlMRS2-4* was relatively more responsive to a low Mg^2+^ environment.

Some of the Mg^2+^ stress or toxicity symptoms appear like Mg^2+^ deficiency phenotype symptoms because free Mg^2+^ causes damage via the peroxidation and blockage of carbohydrate transport and they usually appear on old leaves [2,49]. However, whether Mg^2+^ stress can be diagnosed or manifested as a change in the expression level of the Mg^2+^ transporter is still questionable.

The leaves of tomato seedlings are most sensitive to Mg^2+^ deficiency, followed by roots. The seven Mg^2+^ transporter genes in mature leaves (such as the third expanded leaf L4) were significantly up-regulated under Mg^2+^ deficiency conditions to repair the magnesium ion content in the middle and upper leaves. Similarly, in rice, the Mg^2+^ deficiency symptoms appeared more in the middle leaves [50]. Among them, the most sensitive genes to Mg^2+^ deficiency were *SlMRS2-3* and *SlMRS2-1*. The expression levels of *SlMRS2-3* increased by more than six times in middle and lower leaves (L2–L4) compared with control, and were three times higher than MgT, and the expression level of *SlMRS2-1* in lower leaves (L1–L2) increased by more than six times compared with control and MgT. *SlMRS2-2*, *2-3*, *2-4*, *2-5*, and *2-11* were significantly up-regulated in roots under Mg^2+^ deficiency conditions. Among them, *SlMRS2-4* was expressed significantly higher than other genes and could be selected as a marker of Mg^2+^ deficiency in tomato. The differential expressions of Mg^2+^ transporters have previously been reported in *Arabidopsis*, rice, and corn under Mg^2+^ deficiency. For instance, the expression level of *AtMRS2-4* under Mg^2+^ deficiency was significantly increased in *Arabidopsis*, and peaked at 12 h after treatment [14]; the expression of *AtMRS2-7* was up-regulated under low Mg^2+^ condition [13]; *OsMGT1* in leaves and the leaf sheath of rice was only expressed under Mg^2+^ deficiency [15]; the expression level of *OsMRS2-6* in the young mature leaf of rice was down-regulated with moderate Mg^2+^ deficiency [34]; the expression level of *ZmMGT10* in the maize root system increased and reached the peak at 24 h after low Mg^2+^ treatment [16]; and *ZmMGT12* was down-regulated in the root system and down-regulated in the aboveground part [36].

In this study, the *SlMRS2-2* was highly expressed in the root system of tomato seedlings subjected to excessive Mg^2+^ stress. In addition, *SlMRS2-2*, *2-3*, *2-4*, and *2-I* were more sensitive to high Mg^2+^ concentration. Studies have also shown that *AtMRS2-4*, a homologous protein of *SlMRS2-4*, was affected by excessive Mg^2+^ [18], but the response of *SlMRS2-3* and *SlMRS2-4* to Mg^2+^ toxicity was generally lower than that of Mg^2+^ deficiency. Although the expression level of *SlMRS2-I* in various tissues was significantly down-regulated under Mg^2+^ toxicity, however, its expression level was low and hard to detect. Therefore, it is considered that *SlMRS2-2* in the root can be used as a sign of tomato being subjected to Mg^2+^ stress.

In summary, seven CorA/MRS2/ALR type Mg^2+^ transporter genes were identified and confirmed in tomato in this study. Furthermore, their physicochemical properties, gene structure, conserved motifs, and cis-acting elements were detected. This study might provide fundamental data and clues to functionally characterize the Mg^2+^ transporters in tomato.

## 4. Materials and Methods

### 4.1. Identification of Magnesium Transporters

The peptide sequences of MRS2/MGTs of *Arabidopsis*, maize, and rice were attained by using TAIR (https://www.arabidopsis.org/, accessed on 5 May 2023), MaizeGDB (https://maizegdb.org/, accessed on 5 May 2023) and RGAP (http://rice.plantbiology.msu.edu/, accessed on 5 May 2023) databases, respectively. These sequences were used as the queries to search for the candidate *SlMRS2* genes via blastp (E ≤ 1 × 10−10) in a local tomato genetic database using the sequence downloaded from Phytozome (https://phytozome.jgi.doe.gov/pz/portal.html, accessed on 5 May 2023) and National Center Biotechnology Information (NCBI, https://www.ncbi.nlm.nih.gov/, accessed on 5 May 2023) [51]. The putative *SlMRS2* genes were further confirmed by Pfam (http://pfam.xfam.org/, accessed on 5 May 2023) [52] and Smart (http://smart.embl-heidelberg.de/ accessed on 5 May 2023) to verify the CorA domain (PF01544). Based on the peptide sequences of *SlMRS2*, the molecular mass and isoelectric point were calculated using BioXM 2.6, then the online program TMHMM Server v.2.0 (http://www.cbs.dtu.dk/services/TMHMM/, accessed on 5 May 2023) was used to predict whether the proteins have 2 transmembrane domains, and the WoLF PROST online tools (https://www.genscript.com/wolf-psort.html?src=leftbar, accessed on 5 May 2023) were employed to predict the subcellular location of Mg^2+^ transporters.

### 4.2. Phylogenetic Analysis and Gene Distribution

Multiple peptide sequence alignment was conducted using software DNAMAN and aligned by clustalW. Then, the phylogenetic tree was constructed by Mega X with the neighbor-joining method, with 1000 bootstrap replications used to ensure confidence [53], and the ITOL online program was used to organize the phylogenetic tree (https://itol.embl.de/, accessed on 5 May 2023). Chromosome length, gene distribution density, and collinearity analysis were carried out using the software TBtools and the MCScanX [54].

### 4.3. Sequence Analysis and Protein Quaternary Structure

The distribution of the coding region on the genomic sequence was analyzed with the gene structure display server (GSDS) tool (http://gsds.cbi.pku.edu.cn/, accessed on 5 May 2023) [55]. Conserved motifs were extracted using the MEME (http://meme-suite.org/tools/meme, accessed on 5 May 2023) [56]. The online program Plant cis-acting regulatory DNA elements (https://www.dna.affrc.go.jp/PLACE/?action=newplace, accessed on 5 May 2023) was used to search for the cis-acting elements [57]. The model structures of Mg^2+^ transporters were built using the SWISS-MODEL server (https://swissmodel.expasy.org/interactive, accessed on 5 May 2023) [58] and then the software PyMOL was applied to construct the three-dimensional structures of proteins [59].

### 4.4. Plant Materials, Growth Conditions, and Treatments

Qianxi, a commercial hybrid tomato, was used in this study. The seedlings were cultivated in nutrient solution in the plant incubator under the condition of 25 °C/16 h light and 16 °C/8 h dark. The seedlings at the four-leaf stage were transferred to double deionized water for a week to eliminate background Mg^2+^ in the seedling and were then placed into the Yamazaki tomato nutrient solution with 0 mM Mg^2+^ (Mg deficiency; MgD), 1 mM Mg^2+^ (control; CK), and 5 mM Mg^2+^ (Mg toxicity; MgT). The Mg treatments were applied in a completely randomized design with three replicates for each treatment. The composition of nutrient solutions was as follows: Ca(NO_3_)_2_·4H_2_O 354 mg/L, KNO_3_ 404 mg/L, NH_4_H_2_PO_4_ 77 mg/L, Na_2_Fe-EDTA 25 mg/L, H_3_BO_3_ 2.13 mg/L, MnSO_4_·4H_2_O 2.86 mg/L, ZnSO_4_·7H_2_O 0.22 mg/L, CuSO_4_·5H_2_O 0.08 mg/L, (NH_4_)_6_Mo_7_O_2_·4H_2_O 0.02 mg/L [60]. The pH of the nutrient solution was adjusted to 6.5 and was replaced every two days during the experiment.

To detect the temporal expression of *SlMRS2* genes, the roots, leaves, and stems of seedlings were sampled with three biological replications at 0, 6, 12, 24, 48, and 96 h after treatment. To detect the response of gene expressions to different Mg^2+^ statuses, all tested tissues, including leaves (subdivided into L1–L6 according to the age of leaves), stems, roots, and cotyledons, were sampled with three biological replications after 144 h of Mg treatment. All samples were immediately frozen in liquid nitrogen and stored at −80 °C until use.

### 4.5. Gene Cloning

The cDNA sequences of putative *SlMRS2* genes obtained by alignment and functional domain detection were amplified using gene-specific primers (Table S1) with the high-fidelity enzyme PrimerSTAR GXL DNA Polymerase (Takara Bio Inc., Shiga, Japan). Total RNA was extracted using RNAprep Pure Plant Plus Kit (TIANGEN) and reverse transcribed to cDNA with FastKing RT Kit (with gDNase) (TIANGEN). PCR was performed in a 20 μL system in a PCR instrument (Eppendorf). The PCR products were ligated with the pUCm-T vector.

### 4.6. Salmonella typhimurium Mutant Complementation Assay

After sequencing, the *SlMRS2* genes were cut with restriction enzymes and inserted into the pTrc99A vector with DNA ligase (Takara Bio Inc., Shiga, Japan). Recombinant plasmids were transferred into *Salmonella typhimurium* strain MM281 lacking Mg^2+^ transporter genes (CorA/MgtA/MgtB). The experiment was conducted as described previously [14,61]. The bacteria strain MM281 transferred with empty pTrc99A was used as a negative control, and the strain MM1927 (wild type) was used as a positive control in both experiments. For functional complementation analysis, bacteria were cultured on the N-minimal solid medium with different concentrations of Mg^2+^ (0.01, 0.1, 0.5, 1, 2, 5, 10, and 20 mM) at 37 °C for 48 h. The growth rates of transgenic strains were performed in the N minimal medium with Mg^2+^ (0.1, 0.5, 1, and 10 mM) and antibiotics. The initial concentration of cells was adjusted to an OD_600_ of 0.001. The media were incubated at 37 °C, with 180 r/min rotation, and the optical density was measured every 2 h for 30 h.

### 4.7. The Expression Analysis and Quantitative Real-Time PCR

Digital expression analysis was conducted using the RPKM values of the Mg^2+^ transporter gene in different tissues from 2 tomato varieties (LA1589 and LA4345), and the data were downloaded from the Tomato Functional Genomics Database (http://ted.bti.cornell.edu/cgi-bin/TFGD/digital/home.cgi/ accessed on 2 May 2023). TBtools was employed to draw a gene expression heatmap. Total RNA was extracted using RNAprep Pure Plant Plus Kit (TIANGEN) and reverse transcription was performed with FastKing RT Kit (with gDNase) (TIANGEN). Then, the cDNA was used for quantitative real-time PCR with TB Green Premix Ex Taq^TM^ II (Tli RNaseH Plus) (Takara Bio Inc., Shiga, Japan) in AB7500 (Thermo) using gene-specific primers (Table S2). Three technical repeats were performed for each biological sample. The gene *SlActin* was used as the internal reference, and the relative expression was calculated using 2−∆Ct or 2−∆∆Ct [62].

## Figures and Tables

**Table 1:** The predicted information of SlMRS2 genes in tomato.

Code	Gene Symbol	Gene Locus	Gene Name	Gene Length (bp)	Protein Length (aa)	pI	MW (kDa)	Chromosome	Chromosome Location	Subcellular Localization	TM
1	LOC101257182	Solyc06g068490	SlMRS2-1	5655	461	5.01	52.4	6	42564308~42570140	plas	2
2	LOC101245882	Solyc09g065920	SlMRS2-2	4759	377	4.79	42.53	9	64439955~64444758	chlo	2
3	LOC101261964	Solyc01g106900	SlMRS2-3	8358	498	4.56	54.98	1	94503194~94511499	chlo	2
4	LOC101247864	Solyc01g103890	SlMRS2-4	6520	432	5.53	49.12	1	92285408~92291894	nucl/plas	2
5	LOC101267022	Solyc05g012220	SlMRS2-5	5349	415	4.8	46.84	5	5498595~5506278	plas	2
6	LOC101267965	Solyc11g066660	SlMRS2-11	8959	447	6.09	50.37	11	52722376~52732626	plas	2
7	LOC101259511	Solyc03g005390	SlMRS2-I	10485	395	4.72	44.2	3	224633~235094	vacu	2

MW: molecular weight, pI: isoelectric point, TM: transmembrane domain, chlo: chloroplast membrane, plas: plasma membrane, vacu: vacuole membrane, nucl: nucleus.

## Data Availability

Not applicable.

## Supplementary Materials

The following supporting information can be downloaded at: https://www.mdpi.com/article/10.3390/plants12132512/s1, Table S1: The primers used for the cDNA amplification; Table S2: The primers used for qRT-PCR.; Figure S1. Transmembrane domains of Mg^2+^ transporters in tomato.
