# Supplementary material for: Identification and Expression of the CorA/MRS2/ALR Type Magnesium Transporters in Tomato
Source: Plants (Basel). 2023 Jun 30;12(13):2512. doi: 10.3390/plants12132512 (PMC10347230; doi:10.3390/plants12132512)
Supplement: Supplementary file 1 [file plants-12-02512-s001.zip › plants-2451019-supplementary.pdf]

Table S1. The primers used for the cDNA amplification.

gene name	Primer	sequence	amplicon length
SlMRS2-1	Forward	ATTGGATGACCAGCGCTC	1391 bp
	Reverse	GAATCTACAGTGGCATCAA	
SlMRS2-2	Forward	GTATGAAAACAGGGGTATCC	1139 bp
	Reverse	GATTTAAGATCCAACAAGACCC	
SlMRS2-3	Forward	TATGAGGGGCGTTACTCC	1500 bp
	Reverse	GTTCACTCGAGCAATTGC	
SlMRS2-4	Forward	GATGGGGAAGAATCAATTC	1305 bp
	Reverse	GGATTTCATGATCCAAGC	
SlMRS2-5	Forward	ATGGCAGAAGAAAATGGTC	1252 bp
	Reverse	CTGTTTACAGTGGAAGAAG	
SlMRS2-11	Forward	ATGGCGGCAAATGCTCTG	1344 bp
	Reverse	TTACAGTATTTTCCTCGTTC	
SlMRS2-I	Forward	GATAATPGGGTCGAGAAGGAG	1195 bp
	Reverse	AACTCATGATCCAACAAGCC	

Table S2. The primers used for qRT-PCR

gene name	Primer	sequence	amplicon length
SlMRS2-1	Forward	TGCCACTGTAGATTCTCAACAGCAT	149 bp
	Reverse	AGTAGATCCATCACCTTTTCTGGC	
SlMRS2-2	Forward	TTGTTTGACAGTGTACTCCTTGGTG	138 bp
	Reverse	ATTGATAGGAAAACGGATGCAGAAG	
SlMRS2-3	Forward	TTGCTCGAGTGAACAGAGAGATTTG	134 bp
	Reverse	ATTTCGGAGAATCGGATACACTTGT	
SlMRS2-4	Forward	CACACAGAGGGGTTATTTTGGCC	139 bp
	Reverse	TTCGTAAACCTGGACACAATAGAGG	
SlMRS2-5	Forward	TAAGCACAAGAAACTTCTTCCACTGT	159 bp
	Reverse	GATAGGCAAAAGCAAAAGTTCTGTCT	
SlMRS2-11	Forward	TGATTCTACAGGTTGGAACATTTTG	115 bp
	Reverse	GTTGTAAGCCAGAATGCAAACACAT	
SlMRS2-I	Forward	TATAAAGGGCTTGTTGGATCATGAG	156 bp
	Reverse	AGACTATGGTCATTCACAGCTTTGG	
SlActin	Forward	GAAATAGCATAAGATGGCAGACG	159 bp
	Reverse	ATACCCACCATCACACCAGTAT	

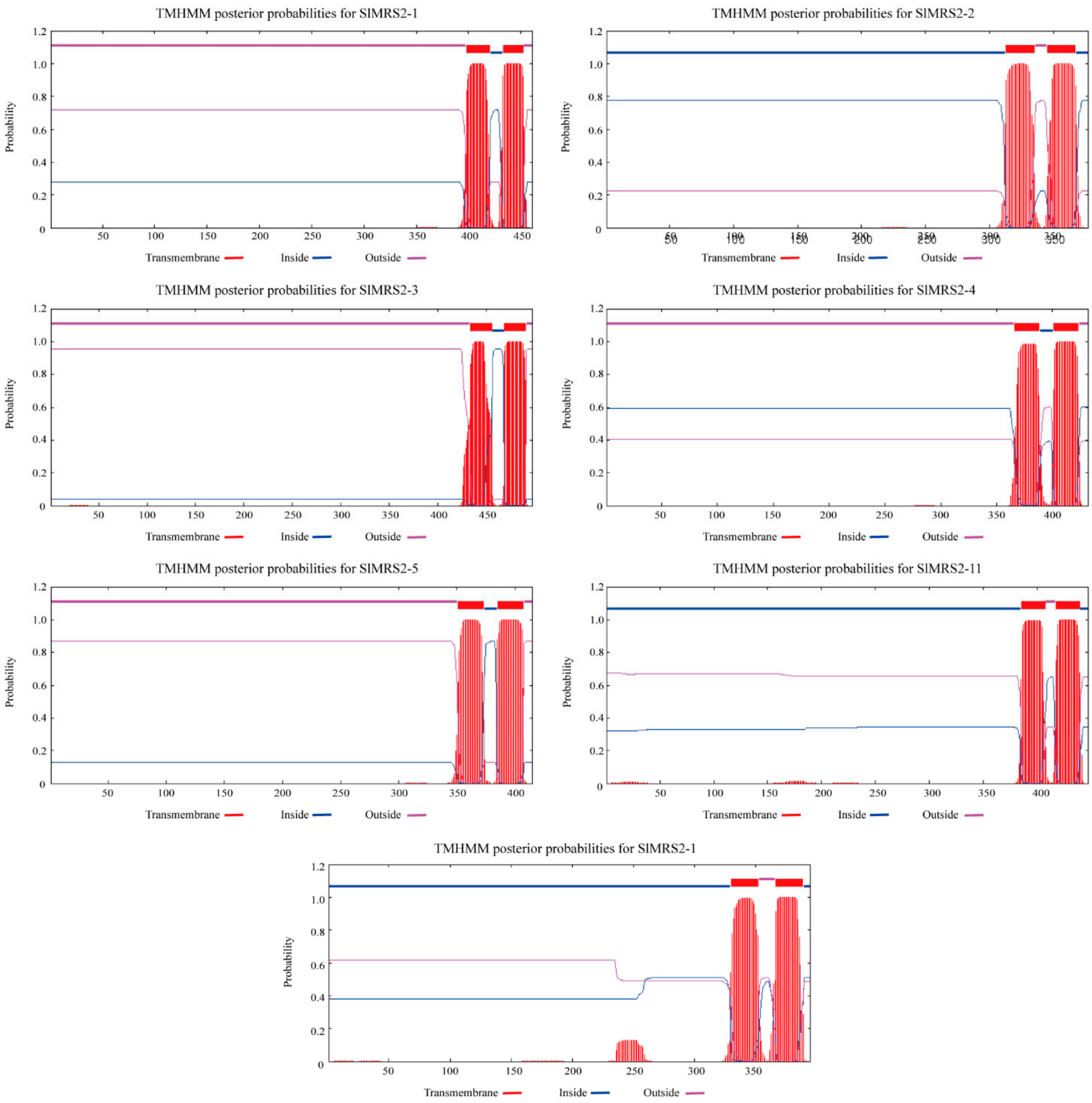

Figure S1. Transmembrane domains of Mg^{2+} transporters in tomato. The transmembrane domains were predicted tusing the online program TMHMM Server v.2.0 and colored in red.
